# Investigation on performance of multiple AI-based auto-contouring systems in organs at risks (OARs) delineation

**DOI:** 10.1007/s13246-024-01434-9

**Published:** 2024-09-02

**Authors:** Young Woo Kim, Simon Biggs, Elizabeth Claridge Mackonis

**Affiliations:** 1 Department of Radiation Oncology, Chris O’Brien Lifehouse, Sydney, NSW Australia; 2 Institute of Medical Physics, School of Physics, University of Sydney, Sydney, NSW Australia; 3 Radiotherapy AI, Sydney, NSW Australia

**Keywords:** Radiation therapy planning, Auto contouring, Auto segmentation, Artificial Intelligence (AI)

## Abstract

Manual contouring of organs at risk (OAR) is time-consuming and subject to inter-observer variability. AI-based auto-contouring is proposed as a solution to these problems if it can produce clinically acceptable results. This study investigated the performance of multiple AI-based auto-contouring systems in different OAR segmentations. The auto-contouring was performed using seven different AI-based segmentation systems (Radiotherapy AI, Limbus AI version 1.5 and 1.6, Therapanacea, MIM, Siemens AI-Rad Companion and RadFormation) on a total of 42 clinical cases with varying anatomical sites. Volumetric and surface dice similarity coefficients and maximum Hausdorff distance (HD) between the expert’s contours and automated contours were calculated to evaluate their performance. Radiotherapy AI has shown better performance than other software in most tested structures considered in the head and neck, and brain cases. No specific software had shown overall superior performance over other software in lung, breast, pelvis and abdomen cases. Each tested AI system was able to produce comparable contours to the experts’ contours of organs at risk which can potentially be used for clinical use. A reduced performance of AI systems in the case of small and complex anatomical structures was found and reported, showing that it is still essential to review each contour produced by AI systems for clinical uses. This study has also demonstrated a method of comparing contouring software options which could be replicated in clinics or used for ongoing quality assurance of purchased systems.

**Supplementary Information:**

The online version contains supplementary material available at 10.1007/s13246-024-01434-9.

## Introduction

To create a patient-specific radiotherapy plan, the radiation oncologists (ROs) manually contour the tumour or target region and organs at risk (OARs) on the patient’s computed tomographic (CT) or magnetic resonance (MR) images. The accuracy of the contours is essential as inaccurate contours have the potential to affect the outcome of the treatment. The manual contouring process is time-consuming, and the time taken for manual contouring can vary according to professionals’ abilities and knowledge. It can take several hours to complete contouring for one patient [4]. Previous studies found that manual contouring can take up to 3 h in Head and Neck intensity-modulated radiotherapy (IMRT) planning [9].

These factors can also lead to noticeable delays in treatment, resulting in unwanted treatment outcomes [4]. A previous study found that the increased waiting time for radiotherapy can increase the risk of local recurrence, which can be translated into decreased overall survival rate in some clinical situations [6].

Additionally, the contouring process suffers from large inter- and intra- observer contouring variabilities between professionals [9, 12, 17, 20]. A considerable mean volume variations of about 50% during parotid delineations was found [9]. A study of inter-observers/institutions variability in target and OARs contouring for breast radiotherapy planning found that the overlap between manually contoured structures was low (up to 10%) and the variation between manually contoured volumes had standard deviations up to 60% [17]. Inter-observer variations were also found in radiotherapy planning for other anatomical sites such as cervical cancer radiotherapy [12] and oral cavity cancer radiotherapy [20]. Inter-observer variation has been shown to have a dosimetric impact during radiation therapy planning [17].

The auto-segmentation method has the potential to replace manual contouring. This auto-contouring technique was developed based on the capability of the algorithms to use prior knowledge. In the early stage, the auto-contouring technique had no or minimal capability of using prior knowledge due to limitations on computing power and the limited availability of prior segmentation data. These were low-level segmentation approaches such as intensity thresholding, region growing, and heuristic edge detection [4]. As the computer powers rapidly developed along with a much larger availability of prior knowledge, the auto-contouring developed rapidly, for example, Atlas-based auto-contouring and deep-learning auto segmentation depending on the size of prior knowledge used in the technique.

Deep-learning auto-segmentation is a technique of machine learning where the algorithms learn or get trained to calculate the final contour. This technique uses a multi-layer neural network called convolution neural networks (CNNs) [4, 31]. A large set of pre-contoured data referred to as training data, is passed through the CNNs to train the algorithm and optimise its parameters through the backpropagation algorithm to calculate and create the optimised contour for target structures [16, 31]. The type and performance of deep-learning based auto-segmentation depend on which network structure was used, such as U-Net [24], V-Net (3D version of U-Net) [4] or ResNet [14] and the quality and quantity of training data set [2, 31]. More advanced network structures such as vision transformer (ViT) were introduced [28] and other studies showed ViT performed better than CNNs when both networks were trained on larger datasets [11].

Many studies have compared the performance of in-house AI-based, and atlas-based auto-contouring systems in OAR delineation accuracy in different cancer types such as Head and neck [5], breast [8], and liver [1]. Even though these studies had demonstrated its better performance in OAR contouring and better efficiency over atlas-based auto-contouring, the development and implementation of in-house AI-based auto-contouring can be complex due to challenges such as the required expertise in developing and implementing the programming code and limitations in collecting a large amount of “training” set [26].

In this study, we compared the performance of seven different commercially available AI-based auto contouring systems: Radiotherapy AI (Radiotherapy AI, Sydney, Australia), 2 different versions of Limbus Contour (Limbus AI Inc, Regina, SK, Canada), Therapanacea ART-plan Annotate (Therapanacea, Paris, France), MIM Contour Protégé AI (MIM, Cleveland, USA) Siemens AI-Rad Companion Organs RT (Siemens Healthineers, Erlangen, Germany) and RadFormation AutoContour (RadFormation, New York, USA) in OAR delineation.

## Method

### Clinical dataset

A total of 42 clinical cases (10 head and neck (HN), 10 brain (B), 10 pelvis (PLV), 4 breast (BT), 4 lung (L) and 4 abdomen (ABO) cases) treated at Chris O’Brien Lifehouse between 2019 and 2021 were selected in this study. The patient scans were selected consecutively from the clinical patient scans for each relevant body site. The computed tomographic (CT) images were acquired with the Canon Aquilion LB CT scanner. Different CT scan parameters were used depending on the patient and anatomical site scanned, illustrated in Table 1. Twenty-three organs at risk were delineated by a single expert for each corresponding case, including brain (total number of sample, n = 10), brainstem (n = 19), left eye (n = 12), right eye (n = 12), spinal cord (n = 19), oesophagus (n = 12), optic chiasm (n = 11), left optic nerve (n = 11), right optic nerve (n = 11), left parotid gland (n = 10), right parotid gland (n =9), left submandibular gland (n = 5), right submandibular gland (n = 4), bladder (n = 10), left femoral head (n = 10), right femoral head (n = 10), heart (n = 7), liver (n = 6), left kidney (n = 5), right kidney (n = 5), left lung (n = 9), right lung (n = 9), rectum (n = 10), and stomach (n = 4). During this study, the manual contours of OARs in each case were considered as the reference contours to be compared with automated contours from AI systems.

**Table 1:** CT parameters used for each tested case

Anatomical site	kVp	Exposure (mAs)	Slice Thickness (mm)	Pixel spacing
Head and neck (HN)	135	82–195	3	1.046–1.361
Brain (B)	120	250	1	0.918–1.100
Pelvis (PLV)	135	50–89	2–3	0.906–1.596
Breast (BT)	120	100–193	3	1.074–1.356
Lung (L)	135	52–198	2	0.826–1.169
Abdomen (ABO)	135	72–147	2	1.105–1.500

### AI-based auto-contouring systems

Seven different AI-based segmentation systems were used to delineate the same OARs contoured in each case during this study, Limbus Contour version 1.5 and 1.6, MIM Contour Protégé AI version 1.1.1, Radformation AutoContour version 2.0.19, Radiotherapy AI version RTAI lifehouse-v0.2.0, Siemens AI-Rad Companion Organs RT (AIRC) version VA31A and Therapanacea ART-plan Annotate version 1.10.1. Each AI system uses different network structures to train its model. Limbus Contour [22] and MIM Contour Protégé AI [29] both use CNN based on U-Net structure. Radformation AutoContour [18] uses CNN based on V-Net structure. Siemens AI-Rad Companion Organs RT [15] uses deep image-to-image network (DI2IN). Radiotherapy AI uses an adapted 3D U-Net. The author were unable to identify the network used for Therapanacea ART-plan Annotate.

Radiotherapy AI used clinical data from Chris O’Brien Lifehouse as the training data set for its model. The training data set and the data set used for this study were mutually exclusive. Radiotherapy AI is in the development stage and is not commercially available yet.

### Quantitative evaluation method

The volumetric Dice Similarity Coefficient (DSC), surface Dice Similarity Coefficient (sDSC) and maximum Hausdorff Distance (HD) between manual segmentation and AI-based auto-contouring systems’ segmentation were calculated to quantitatively evaluate the performance of each AI-based auto-contouring software in OAR delineations [25]. The DSC, sDSC and HD were calculated using python script with PlatiPy version 0.4.0 [7]. The volumetric Dice Similarity Coefficient (DSC) calculates the overlap between 2 contoured volumes and is defined as:

$$\begin{aligned} DSC = \frac{2|A|\cap |B|}{|A| + |B|} \end{aligned}$$

Where A is the volume of manual contours and B is the volume of contours delineated by an AI system. The value of the DSC metric varies from 0, which illustrates no overlap between two contours, to 1, which illustrates the complete overlap between two contours.

The surface Dice Similarity Coefficient (sDSC) is a new metric for assessing the segmentation performance introduced by Nikolov et al [21]. This metric calculates the overlap between the two surfaces at a defined tolerance ($\tau$) and is defined as:

$$\begin{aligned} sDSC^{(\tau )}_{A,B}=\frac{|S_A \cap B_{B}^{(\tau )}| + |S_B \cap B_{A}^{(\tau )}|}{|S_A|+|S_B|} \end{aligned}$$

where $S_A$ and $S_B$ are surface of manual contours (A) and AI contours (B) and $B_A$ and $B_B$ are the border regions of manual contours (A) and AI contours (B) respectively. As in radiotherapy, the OAR is contoured slice by slice and the segmentation performance is assessed by the fraction of the surface of the contour which needed to be edited, sDSC has been suggested as a more suitable metric compared to volumetric DSC to assess the segmentation performance as the volumetric DSC weighs all regions where two volumes do not overlap equally and independently of their distance from the surface, and is biased towards OARs which has large volume [21]. Another study showed that sDSC is a better indicator than DSC and HD of the time needed to edit and time saved by using auto contouring systems [27]. The tolerance parameter $\tau$ needs to be set appropriately where variation is clinically acceptable by measuring inter-observer variation in contouring [21]. For this study, $\tau$ value of 0 mm was used for sDSC calculation to evaluate the absolute difference between manual and AI system’s contours and additional sDSC calculations with different $\tau$ values (1,2,3 mm) were performed as previous study by Rhee *et. al* found sDSC with tolerance value of 1, 2, 3 mm are most accurate similarity metrics compared to other metrics used to detect the errors in contour [23].

The maximum Hausdorff Distance (HD) between two contoured volumes to calculate the greatest distance from a point in one contour to the closest point in the other contour based on equation:

$$\begin{aligned} HD(A,B)\ =\ \text {max}(h(A,B),h(B,A)) \end{aligned}$$

Where h(A,B) is the directed Hausdorff distance between A and B. The directed Hausdorff distance is expressed as:

$$\begin{aligned} h(A,B)\ =\ \text {max}_{a\in A} \text {min}_{b\in B}||a-b|| \end{aligned}$$

$||a-b||$ is the Euclidean distance between point a in A and point b in B. The zero HD value represents there is no difference between 2 contours’ shapes but as the HD value increases, the difference between 2 contours’ shapes are increasing.

To ensure a valid comparison, cases with non-identical numbers of data sets were divided into separate groups, ensuring that each set had an equal number of data points when calculating mean DSC, sDSC and HD. For instance, 19 cases were selected for testing in spinal cord segmentation. However, data from RTAI was unavailable for 9 out of the 19 cases, as the RTAI model was exclusively designed for Head and Neck cases at the time of the study.

### Statistical analysis

The statistical difference between each index of DSC and HD for each tested AI-based software was calculated using a suitable type of statistical test between 3 tests, (1) Student’s *t*-test, (2) Welch’s *t*-test and (3) Wilcoxon-Signed Rank test, depending on properties of compared data sets with a p-value lesser than 0.05 indicating significance [26]. The test was automated using an in-house Python script combined with published python packages. The box plots of each data set in each case were created to check if there are any outliers. Then the histogram was created to visually inspect the distribution of data. The Shapiro-Wilk and Q-Q plot tests were used to test the normality of the distribution of each sample. When the data was assumed to be normally distributed, the F-test was used to find whether each compared data set’s variance are equal. The Student’s *t*-test was used in case of equal variance between 2 compared data sets, and the Welch’s *t*-test was used in case of unequal variances between 2 compared data sets. The Wilcoxon-Signed Rank test was used when both compared data sets were not normally distributed and when normally distributed data sets were compared with data sets which were not normally distributed. It was also used to compare two data sets where any one of the data sets or both had outlier data points [13]. The detailed results of statistical test conducted during study can be found in supplementary data A (DSC), B (HD) and C (sDSC).

**Fig. 1:**
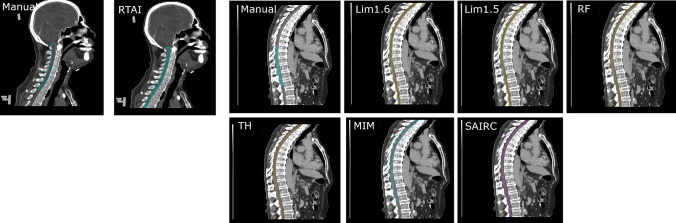
Manual and AI systems’ contour of the spinal cord in Varian Eclipse Treatment Planning system

**Fig. 2:**
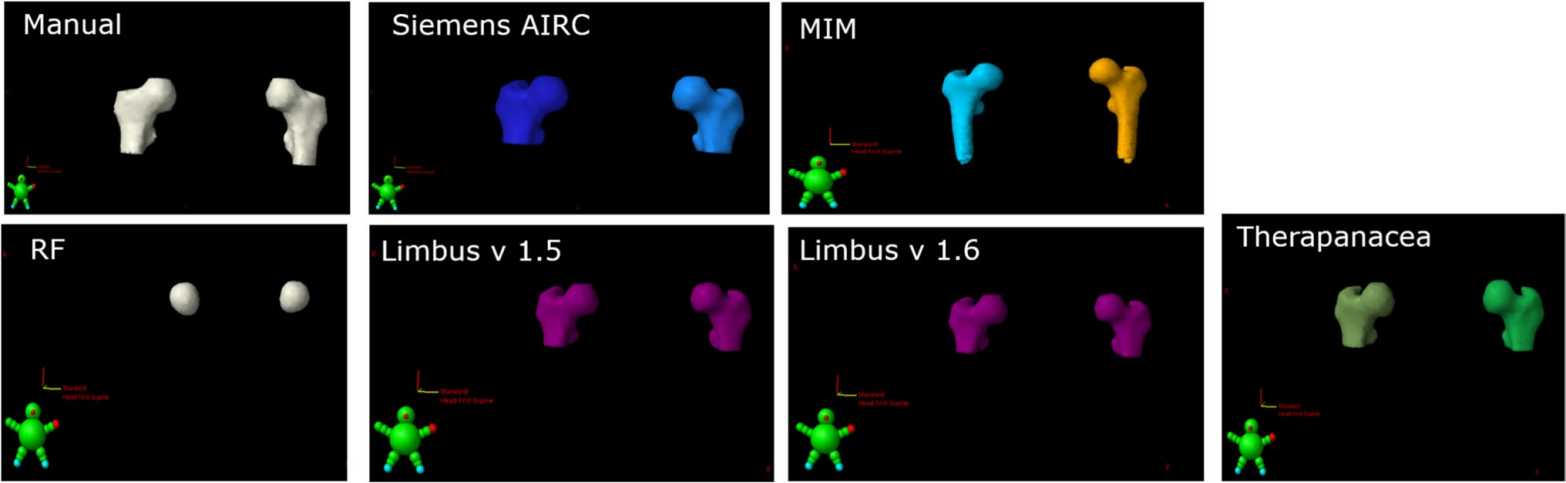
3D representation of manual and AI systems’ contour of both left and right femoral heads in Varian Eclipse Treatment Planning system

**Table 2:** Dice Similarity Coefficient (DSC) values between manual contours and individual automated contours of OARs considered

	Brain (n = 10)			Brainstem (n = 19)			Left eye (n = 12)			Right Eye (n =12)		
	Mean	STD (±)	Range	Mean	STD (±)	Range	Mean	STD (±)	Range	Mean	STD (±)	Range
Limbus AI v1.6	0.960	0.010	0.950–0.984	0.848	0.038	0.749–0.893	0.901	0.039	0.789–0.929	0.905	0.031	0.862–0.954
Limbus AI v1.5	0.960	0.010	0.950–0.984	0.844	0.038	0.740–0.893	0.894	0.038	0.791–0.938	0.896	0.033	0.846–0.947
MIM	0.955	0.007	0.945–0.968	0.806	0.045	0.734–0.877	0.900	0.044	0.782–0.952	0.902	0.027	0.856–0.943
RadFormation	0.961	0.010	0.947–0.984	0.834	0.051	0.744–0.890	0.913	0.044	0.791–0.953	0.911	0.031	0.861–0.967
Radiotherapy AI	0.958	0.013	0.942–0.985	**0.878**	0.039	0.801–0.932	**0.934**	0.047	0.806–0.979	**0.935**	0.034	0.876–0.972
Siemens AIRC	0.955	0.010	0.936–0.967	0.778	0.060	0.663–0.884	0.897	0.041	0.796–0.942	0.899	0.027	0.859–0.953
Therapanacea	**0.962**	0.012	0.949–0.990	0.861	0.035	0.770–0.904	0.910	0.039	0.803–0.958	0.917	0.023	0.868–0.957

Maximum difference	0.007			0.100			0.040			0.039		
	Optic chiasm (set 1 (n = 10))			Left optic nerve (set 1 (n = 10))			Right optic nerve (n = 11)			Spinalcord (set 1 (n =10))		
	Mean	STD (±)	Range	Mean	STD (±)	Range	Mean	STD (±)	Range	Mean	STD (±)	Range
Limbus AI v1.6	0.432	0.185	0.000–0.586	0.536	0.173	0.065–0.675	0.585	0.159	0.154–0.806	0.682	0.107	0.427–0.797
Limbus AI v1.5	0.434	0.107	0.212–0.512	0.536	0.173	0.065–0.675	0.585	0.158	0.154–0.794	0.667	0.112	0.407–0.790
MIM	0.402	0.104	0.232–0.583	0.400	0.134	0.069–0.524	0.472	0.104	0.356–0.703	0.726	0.086	0.563–0.837
RadFormation	**0.483**	0.178	0.000–0.639	0.513	0.129	0.164–0.654	0.551	0.106	0.276–0.703	0.743	0.077	0.595–0.838
Radiotherapy AI	0.483	0.383	0.086–0.941	**0.700**	0.188	0.484–0.942	**0.707**	0.211	0.418–0.931	**0.763**	0.057	0.644–0.840
Siemens AIRC	0.315	0.174	0.000–0.568	0.529	0.166	0.075–0.652	0.512	0.140	0.160–0.639	0.559	0.083	0.442–0.676
Therapanacea	0.467	0.124	0.253–0.599	0.540	0.155	0.111–0.646	0.588	0.143	0.205–0.785	0.748	0.045	0.664–0.826

Maximum difference	0.168			0.300			0.235			0.204		
	Left parotid gland (n = 10)			Right parotid gland (n = 9)			Left submandibular gland (n = 5)			Right submandibular gland (n =4)		
	Mean	STD (±)	Range	Mean	STD (±)	Range	Mean	STD (±)	Range	Mean	STD (±)	Range
Limbus AI v1.6	0.824	0.065	0.718–0.899	0.848	0.045	0.766–0.903	0.850	0.033	0.806–0.883	0.837	0.006	0.832–0.847
Limbus AI v1.5	0.824	0.065	0.718–0.898	0.847	0.045	0.765–0.903	0.847	0.037	0.805–0.890	0.840	0.009	0.831–0.853
MIM	0.806	0.073	0.671–0.879	0.813	0.047	0.735–0.864	0.794	0.088	0.641–0.850	0.736	0.127	0.549–0.825
RadFormation	0.817	0.059	0.701–0.874	0.812	0.071	0.693–0.883	0.843	0.029	0.800–0.880	0.845	0.010	0.832–0.856
Radiotherapy AI	**0.853**	0.066	0.742–0.923	**0.857**	0.062	0.762–0.929	**0.902**	0.075	0.783–0.954	**0.925**	0.056	0.841–0.960
Siemens AIRC	0.797	0.075	0.681–0.878	0.823	0.061	0.720–0.901	0.853	0.048	0.772–0.896	0.852	0.033	0.808–0.888
Therapanacea	0.831	0.066	0.722–0.896	0.854	0.047	0.770–0.910	0.863	0.024	0.825–0.885	0.838	0.030	0.805–0.867

Maximum difference	0.056			0.045			0.107			0.189		
	Oesophagus (set 1 (n = 6))			Oesophagus (set 3 (n = 2))			Optic chiasm (set 2) **			Left optic nerve (set 2) **		
	Mean	STD (±)	Range	Mean	STD (±)	Range	Mean			Mean		
Limbus AI v1.6	0.786	0.100	0.602–0.869	0.637	0.142	0.536–0.737	0.442			0.712		
Limbus AI v1.5	****	****	****	****	****		0.408			0.732		
MIM	***	***	***	***	***		0.403			0.577		
RadFormation	0.689	0.108	0.522–0.823	0.593	0.057	0.553–0.634	0.380			0.640		
Radiotherapy AI	**0.828**	0.080	0.693–0.917	**0.714**	0.088	0.652–0.777	**0.851**			***		
Siemens AIRC	***	***	***	0.621	0.202	0.479–0.764	***			0.379		
Therapanacea	0.811	0.110	0.613–0.896	0.608	0.167	0.490–0.727	0.364			**0.803**		
Maximum difference	0.139			0.121			0.487			0.423		

Bold underline values indicate the highest DSC values

*No available model from AI system

**Result from only 1 tested case

***No contours produced by AI system

****Corresponding organ was not included to be contour by AI system in template

**Table 3:** Dice Similarity Coefficient (DSC) values between manual contours and individual automated contours of OARs considered

	Left femoral head (set 1 (n =5))			Left femoral head (set 2 (n = 5))			Right femoral head (set 1 (n = 5))			Right femoral head (set 2 (n = 5))		
	Mean	STD (±)	Range	Mean	STD (±)	Range	Mean	STD (±)	Range	Mean	STD (±)	Range
Limbus AI v1.6	0.813	0.092	0.723–0.941	0.936	0.026	0.903–0.967	0.815	0.088	0.724 - 0.943	0.910	0.064	0.797–0.947
Limbus AI v1.5	0.813	0.092	0.723–0.940	0.936	0.026	0.903–0.967	0.818	0.088	0.738–0.943	0.910	0.064	0.797–0.948
MIM	*	*	*	0.810	0.042	0.731–0.849	*	*	*	0.784	0.078	0.646–0.873
RadFormation	0.385	0.061	0.338–0.489	0.452	0.092	0.299–0.549	0.382	0.054	0.339–0.475	0.346	0.206	0.000–0.475
Siemens AIRC	0.826	0.089	0.748–0.956	0.918	0.019	0.895–0.938	**0.835**	0.079	0.762–0.957	0.903	0.040	0.834–0.936
Therapanacea	**0.826**	0.089	0.731–0.950	**0.944**	0.030	0.910–0.969	0.824	0.086	0.758–0.953	**0.913**	0.089	0.758–0.978

Maximum difference	0.442			0.492			0.452			0.567		
	Left kidney (set 1 (n = 4))			Right kidney (set 1 (n = 4))			Heart (n = 7)			Spinalcord (set 2 (n = 9))		
	Mean	STD (±)	Range	Mean	STD (±)	Range	Mean	STD (±)	Range	Mean	STD (±)	Range
Limbus AI v1.6	**0.926**	0.013	0.915–0.945	0.936	0.028	0.909–0.972	0.911	0.031	0.867–0.958	0.559	0.235	0.000–0.770
Limbus AI v1.5	0.926	0.016	0.911–0.948	**0.943**	0.021	0.915–0.965	0.911	0.031	0.868–0.958	0.553	0.237	0.000–0.770
MIM	0.913	0.026	0.887–0.948	0.927	0.016	0.914–0.949	0.909	0.025	0.875–0.940	0.597	0.127	0.401–0.760
RadFormation	0.910	0.042	0.857–0.959	0.928	0.039	0.889–0.972	**0.911**	0.033	0.871–0.963	0.587	0.135	0.394–0.751
Siemens AIRC	0.904	0.043	0.845–0.947	0.915	0.034	0.878–0.950	0.895	0.043	0.841–0.959	0.559	0.156	0.329–0.849
Therapanacea	0.915	0.036	0.873–0.959	0.935	0.035	0.900–0.973	0.902	0.036	0.854–0.966	**0.579**	0.176	0.294–0.806

Maximum difference	0.022			0.028			0.016			0.044		
	Left lung (set 1 (n = 8))			Right lung (set 1 (n = 8))			Stomach (n = 4)			Oesophagus (set 2 (n = 4))		
	Mean	STD (±)	Range	Mean	STD (±)	Range	Mean	STD (±)	Range	Mean	STD (±)	Range
Limbus AI v1.6	0.974	0.004	0.968–0.980	0.978	0.008	0.962–0.990	0.849	0.089	0.728–0.931	0.761	0.055	0.697–0.831
Limbus AI v1.5	0.973	0.005	0.966–0.980	0.977	0.007	0.966–0.989	**0.849**	0.089	0.728–0.931	0.780	0.033	0.757–0.828
MIM	0.963	0.012	0.942–0.975	0.968	0.008	0.954–0.977	***	***	***	0.722	0.047	0.654–0.762
RadFormation	0.965	0.015	0.944–0.981	0.972	0.015	0.950–0.989	0.762	0.173	0.544–0.933	0.681	0.122	0.519–0.813
Siemens AIRC	0.963	0.022	0.910–0.977	0.973	0.007	0.962–0.981	***	***	***	0.753	0.136	0.552–0.851
Therapanacea	**0.976**	0.009	0.963–0.989	**0.979**	0.009	0.963–0.989	0.832	0.079	0.721–0.907	**0.784**	0.046	0.741–0.842

Maximum difference	0.014			0.011			0.087			0.103		
	Liver (set 1 (n = 4))			Liver (set 2 (n = 2))			Rectum (set 1 (n = 5))			Rectum (set 2 (n = 5))		
	Mean	STD (±)	Range	Mean	STD (±)	Range	Mean	STD (±)	Range	Mean	STD (±)	Range
Limbus AI v1.6	**0.958**	0.005	0.952–0.964	**0.956**	0.012	0.948–0.965	0.668	0.164	0.424–0.873	0.815	0.068	0.720–0.902
Limbus AI v1.5	0.956	0.008	0.945–0.963	****	****	****	0.667	0.164	0.423–0.872	**0.815**	0.068	0.721–0.902
MIM	0.943	0.008	0.938–0.954	0.943	0.014	0.933–0.952	*	*	*	0.766	0.066	0.673–0.865
RadFormation	0.942	0.006	0.936–0.951	0.950	0.012	0.941–0.959	0.639	0.161	0.407–0.842	0.688	0.259	0.235–0.885
Siemens AIRC	0.943	0.008	0.931–0.949	0.940	0.011	0.932–0.948	0.589	0.174	0.375–0.773	0.779	0.068	0.675–0.850
Therapanacea	0.948	0.010	0.940–0.962	0.955	0.013	0.946–0.964	**0.679**	0.173	0.447–0.811	0.796	0.096	0.667–0.883

Maximum difference	0.015			0.016			0.090			0.127		
	Bladder (set 1 (n = 5))			Bladder (set 2 (n = 5))			Left kidney (set 2) **	Right kidney (set 2) **	Left lung (set 2) **	Right lung (set 2) **		
	Mean	STD (±)	Range	Mean	STD (±)	Range	Mean	Mean	Mean	Mean		
Limbus AI v1.6	0.959	0.020	0.924–0.972	**0.962**	0.006	0.954–0.968	0.920	0.922	0.880	0.915		
Limbus AI v1.5	0.959	0.020	0.924–0.972	0.962	0.006	0.955–0.968	****	****	****	****		
MIM	*	*	*	0.948	0.013	0.931–0.964	*	*	***	***		
RadFormation	0.948	0.041	0.876–0.971	0.908	0.104	0.722–0.960	**0.944**	**0.936**	**0.900**	**0.931**		
Siemens AIRC	0.920	0.051	0.831–0.961	0.922	0.034	0.881–0.950	0.930	0.924	0.879	0.920		
Therapanacea	**0.960**	0.018	0.931–0.978	0.956	0.006	0.948–0.965	0.938	0.933	0.892	0.930		
Maximum difference	0.040			0.053			0.024	0.014	0.021	0.016		

Bold underline values indicate the highest DSC values

*No available model from AI system

**Result from only 1 tested case

***No contours produced by AI system

****Corresponding organ was not included to be contour by AI system in template

**Table 4:** Maximum Hausdorff Distance (HD) values between manual contours and individual automated contours of OARs considered

	Brain (n = 10)			Brainstem (n = 19)			Left eye (n = 12)			Right eye (n =12)		
	Mean (mm)	STD (±mm)	Range (mm)	Mean (mm)	STD (±mm)	Range (mm)	Mean (mm)	STD (±mm)	Range (mm)	Mean (mm)	STD (±mm)	Range (mm)
Limbus AI v1.6	11.4	1.2	9.8–13.6	8.2	3.7	4.4–18.5	3.0	1.1	2.1–6.2	3.0	0.9	1.4–4.4
Limbus AI v1.5	11.4	1.2	9.8–13.6	8.4	3.7	4.4–18.5	3.1	1.2	2.1–6.5	3.1	0.9	1.5–4.4
MIM	11.5	1.0	9.9–13.1	9.5	2.5	5.9–16.1	3.0	1.3	2.0–6.7	3.3	1.0	2.0–5.4
RadFormation	11.2	1.2	9.4–13.3	8.1	3.1	4.5–15.3	3.0	1.2	1.8–6.6	2.8	0.8	1.4–4.1
Radiotherapy AI	11.8	1.4	10.0–15.0	**6.4**	3.1	3.0–14.1	**1.0**	1.3	1.1–5.9	**2.4**	0.9	1.1–3.6
Siemens AIRC	11.3	1.2	9.8–13.0	11.9	3.5	6.1–17.8	3.3	1.2	2.1–6.3	3.2	0.9	1.4–4.4
Therapanacea	**11.0**	1.2	8.8–13.1	7.7	2.8	5.2–15.0	2.8	1.1	1.6–5.8	2.7	0.8	1.5–4.3

Maximum difference	0.9			5.5			2.3			0.8		
	Optic chiasm (set 1 (n = 9))			Optic chiasm (set 2 (n = 2)			Left optic nerve (set 1 (n = 10))			Right optic nerve (n = 11)		
	Mean (mm)	STD (±mm)	Range (mm)	Mean (mm)	STD (±mm)	Range (mm)	Mean (mm)	STD (±mm)	Range (mm)	Mean (mm)	STD (±mm)	Range (mm)
Limbus AI v1.6	**5.6**	0.7	4.7–7.0	8.2	5.5	4.3–12.1	5.0	1.0	3.5–7.1	5.2	1.5	3.0–7.7
Limbus AI v1.5	6.5	1.1	4.2–8.1	8.0	2.6	6.2–9.8	5.0	1.0	3.5–7.1	5.2	1.5	3.0–7.7
MIM	8.1	1.8	4.8–10.1	7.2	3.1	5.0–9.4	6.6	1.4	4.2–8.4	5.8	0.8	4.5–7.2
RadFormation	6.5	1.8	4.6–10.9	5.6	0.0		6.8	1.5	4.6–9.5	6.8	2.1	2.9–9.6
Radiotherapy AI	7.3	4.5	1.4–12.3	**2.0**	0.1	1.9–2.1	**3.3**	2.1	1.0–7.5	**2.8**	1.5	1.0–5.6
Siemens AIRC	10.7	3.2	6.9–16.4	***	***	***	6.1	1.7	2.8–8.4	7.1	2.7	4.2–13.8
Therapanacea	9.3	1.0	8.0–11.2	10.2	1.1	9.4–11.0	4.3	1.2	3.0–7.1	4.3	1.3	3.1–7.4

Maximum difference	5.2			8.2			3.5			4.4		
	Left parotid gland (n = 10)			Right parotid gland (n = 9)			Left submandibular gland (n = 5)			Right submandibular gland (n =4)		
	Mean (mm)	STD (±mm)	Range (mm)	Mean (mm)	STD (±mm)	Range (mm)	Mean (mm)	STD (±mm)	Range (mm)	Mean (mm)	STD (±mm)	Range (mm)
Limbus AI v1.6	14.1	6.8	1.3–24.8	14.4	6.1	6.4–25.5	5.4	1.1	3.9–6.5	6.3	2.0	4.4–9.1
Limbus AI v1.5	14.1	6.8	1.3–24.8	14.4	6.1	6.4–25.5	5.3	1.0	3.9–6.5	6.1	1.8	4.4–8.6
MIM	12.0	4.3	3.3–19.3	11.9	2.4	9.1–15.1	6.6	2.2	4.4–10.3	8.0	0.9	7.4–9.3
RadFormation	14.0	5.3	6.6–23.2	13.1	4.8	9.4–24.9	6.6	2.1	3.2–8.9	6.3	1.2	4.8–7.7
Radiotherapy AI	**10.9**	6.0	1.3–21.3	**11.0**	5.1	6.1–23.2	**3.9**	3.1	1.7–8.8	**3.1**	2.5	1.7–6.9
Siemens AIRC	15.7	7.3	2.7–29.1	15.7	8.5	9.4–37.2	5.4	2.3	3.3–9.4	5.6	0.7	4.6–6.1
Therapanacea	13.2	6.7	1.3–21.2	11.9	3.0	8.1–18.2	5.2	0.9	3.9–6.5	5.7	2.1	4.1–8.7

Maximum difference	4.8			4.7			2.8			4.9		
	Oesophagus (set 1 (n = 6))			Oesophagus (set 3 (n = 2))			Spinalcord (set 1 (n =10))			Left optic nerve (set 2) **		
	Mean (mm)	STD (±mm)	Range (mm)	Mean (mm)	STD (±mm)	Range (mm)	Mean (mm)	STD (±mm)	Range (mm)	Mean (mm)		
Limbus AI v1.6	17.1	18.2	3.8–51.7	42.5	45.8	10.2–74.9	48.2	35.8	9.2–119.4	11.5		
Limbus AI v1.5	****	****	****	****	****	****	48.1	35.8	9.5–119.4	11.5		
MIM	***	***	***	***	***	***	43.8	25.5	7.9–90.9	**3.3**		
RadFormation	20.7	14.3	6.6–44.4	**39.7**	31.5	17.4–62.0	**39.3**	24.5	7.9–87.8	9.6		
Radiotherapy AI	**16.4**	18.5	2.5–51.4	41.9	46.5	9.0–74.8	43.6	25.4	7.2–90.4	***		
Siemens AIRC	***	***	***	40.7	49.0	6.1–75.4	43.2	24.5	11.0–89.3	16.7		
Therapanacea	16.8	18.5	3.3–51.9	42.5	46.3	9.7–75.2	43.5	25.1	8.3–89.9	3.8		
Maximum difference	4.3			2.8			8.9			13.4		

Bold underline values indicate the lowest HD values

*No available model from AI system

**Result from only 1 tested case

***No contours produced by AI system

****Corresponding organ was not included to be contour by AI system in template

**Table 5:** Maximum Hausdorff Distance (HD) values between manual contours and individual automated contours of OARs considered

	Bladder (set 1 (n = 5))			Bladder (set 2 (n = 5))			Left femoral head (set 1 (n =5))			Left femoral head (set 2 (n = 5))		
	Mean (mm)	STD (±mm)	Range (mm)	Mean (mm)	STD (±mm)	Range (mm)	Mean (mm)	STD (±mm)	Range (mm)	Mean (mm)	STD (±mm)	Range (mm)
Limbus AI v1.6	8.5	7.7	3.3–22.0	6.3	0.5	6.0–7.2	62.3	35.8	8.6–94.7	10.5	6.8	2.8–20.5
Limbus AI v1.5	8.5	7.7	3.3–22.0	6.3	0.5	6.0–7.2	62.3	35.8	8.6–94.7	10.5	6.8	2.8–20.5
MIM	*	*	*	7.0	1.8	4.9–10.2	*	*	*	97.5	31.7	67.0–149.7
RadFormation	9.5	7.2	4.1–22.0	9.0	8.4	4.6–24.1	118.4	37.4	58.3–150.3	72.9	9.7	57.8–84.1
Siemens AIRC	12.2	12.9	5.7–35.2	15.3	16.6	4.0–44.2	**60.3**	34.9	8.6–90.4	14.2	5.9	6.6–22.8
Therapanacea	**6.3**	1.9	3.9–9.0	**6.2**	1.3	4.4–8.0	61.0	34.7	8.6–92.9	**9.5**	8.3	2.9–20.5

Maximum difference	5.9			9.1			58.1			87.9		
	Left kidney (set 1 (n = 4))			Right kidney (set 1 (n = 4))			Heart (n = 7)			Spinalcord (set 2 (n = 9))		
	Mean (mm)	STD (±mm)	Range (mm)	Mean (mm)	STD (±mm)	Range (mm)	Mean (mm)	STD (±mm)	Range (mm)	Mean (mm)	STD (±mm)	Range (mm)
Limbus AI v1.6	**7.2**	3.8	4.6–12.7	11.9	4.4	8.4–18.0	**16.5**	6.1	9.0–26.0	110.5	68.6	4.3–212.8
Limbus AI v1.5	9.5	3.3	6.3–13.8	8.9	2.7	6.6–12.3	16.5	6.1	9.0–26.0	114.9	72.0	4.3–212.8
MIM	10.9	1.9	8.4–12.5	**8.3**	1.9	5.8–10.4	17.6	6.3	9.1–27.0	**102.7**	69.2	4.4–213.2
RadFormation	11.6	4.2	6.7–16.3	10.1	4.0	5.8–15.0	17.4	5.9	9.8–26.1	104.4	65.8	27.1–213.2
Siemens AIRC	9.9	1.1	8.5–11.2	10.5	3.5	6.4–14.7	17.0	8.0	7.4–30.1	115.1	74.6	4.3–212.4
Therapanacea	11.4	2.7	8.1–13.6	9.5	2.4	7.1–12.4	18.3	5.9	10.0–28.0	119.1	74.3	4.3–213.1

Maximum difference	4.4			3.6			1.8			16.4		
	Left lung (set 1 (n = 8))			Right lung (set 1 (n = 8))			Stomach (n = 4)			Oesophagus (set 2 (n = 4))		
	Mean (mm)	STD (±mm)	Range (mm)	Mean (mm)	STD (±mm)	Range (mm)	Mean (mm)	STD (±mm)	Range (mm)	Mean (mm)	STD (±mm)	Range (mm)
Limbus AI v1.6	19.5	12.3	6.7–38.9	29.6	21.3	8.3–70.0	**22.3**	6.0	13.7–26.8	31.6	18.9	7.4–53.5
Limbus AI v1.5	19.5	12.4	6.7–38.9	29.9	21.3	8.3–70.0	**22.3**	6.0	13.7–26.8	**23.9**	11.0	11.0–36.9
MIM	**17.4**	11.5	7.8–36.7	29.5	20.7	12.1–68.7	***	***	***	24.1	9.5	12.7–35.8
RadFormation	22.9	11.6	10.9–37.2	28.6	21.3	10.1–69.1	41.4	25.1	20.1–77.1	51.8	45.2	17.5–118.3
Siemens AIRC	20.9	12.8	9.6–43.3	28.8	21.9	9.7–70.3	***	***	***	37.3	46.1	5.9–105.8
Therapanacea	17.8	9.6	8.9–36.0	**28.2**	22.2	8.4–70.0	33.4	26.4	15.9–72.7	29.0	7.9	17.6–35.3

Maximum difference	5.5			1.7			19.2			27.9		
	Liver (set 1 (n = 4))			Liver (set 2 (n = 2))			Rectum (set 1 (n = 5))			Rectum (set 2 (n = 5))		
	Mean (mm)	STD (±mm)	Range (mm)	Mean (mm)	STD (±mm)	Range (mm)	Mean (mm)	STD (±mm)	Range (mm)	Mean (mm)	STD (±mm)	Range (mm)
Limbus AI v1.6	29.6	15.5	15.1–51.3	**12.0**	0.0	12.0	34.7	9.3	20.3–45.6	**22.1**	11.5	9.0–36.2
Limbus AI v1.5	32.0	14.7	23.5–54.0	****	****	****	34.6	9.3	20.3–45.6	**22.1**	11.5	9.0–36.2
MIM	31.4	12.6	16.5–43.8	18.3	4.6	15.0–21.6	*	*	*	35.5	17.7	14.5–57.8
RadFormation	39.4	18.0	24.9–64.3	13.8	1.7	12.6–15.0	**33.7**	7.2	21.2–39.7	33.6	18.3	12.3–59.4
Siemens AIRC	**25.2**	8.0	17.2–35.6	16.3	0.0	16.3	52.1	11.5	36.7–65.4	29.4	13.0	15.6–44.5
Therapanacea	32.9	17.1	12.9–53.6	**12.0**	0.0	12.0	37.7	14.3	24.5–53.4	35.9	18.2	15.0–59.1

Maximum difference	14.1			6.3			18.4			13.8		
	Right femoral head (set 1 (n = 5))			Right femoral head (set 2 (n = 5))			Left kidney (set 2) **	Right kidney (set 2) **	Left lung (set 2) **	Right lung (set 2) **		
	Mean (mm)	STD (±mm)	Range (mm)	Mean (mm)	STD (±mm)	Range (mm)	Mean (mm)	Mean (mm)	Mean (mm)	Mean (mm)		
Limbus AI v1.6	61.5	37.4	3.9–94.7	57.6	91.0	10.7–219.4	7.4	10.3	47.7	33.1		
Limbus AI v1.5	61.3	37.4	3.9–93.5	57.6	91.0	10.7–219.4	**** $^{1}$	****	****	****		
MIM	*	*	*	113.3	54.7	64.9–215.2	*	*	***	***		
RadFormation	119.1	37.0	62.4–152.8	106.0	62.9	73.1–218.3	**6.6**	8.8	**45.5**	**32.1**		
Siemens AIRC	**58.1**	35.6	4.6–92.6	60.7	88.9	13.1–218.8	7.2	**8.6**	48.6	33.1		
Therapanacea	62.9	38.5	3.8–90.9	**56.4**	91.8	2.3–218.6	7.2	**8.6**	46.9	33.1		
Maximum difference	61.0			56.9			0.8	1.7	3.0	0.9		

Bold underline values indicate the lowest HD values

*No available model from AI system

**Result from only 1 tested case

***No contours produced by AI system

****Corresponding organ was not included to be contour by AI system in template

**Table 6:** Surface Dice Similarity Coefficient (sDSC) values between manual contours and individual automated contours of OARs considered

	Brain (n = 10)			Brainstem (n = 19)			Left eye (n = 12)			Right eye (n =12)		
	Mean	STD (±)	Range	Mean	STD (±)	Range	Mean	STD (±)	Range	Mean	STD (±)	Range
Limbus AI v1.6	0.344	0.172	0.818–0.225	0.427	0.085	0.607–0.320	0.524	0.135	0.750–0.195	0.530	0.152	0.774–0.365
Limbus AI v1.5	0.344	0.172	0.818–0.225	0.421	0.082	0.596–0.321	0.501	0.139	0.748–0.197	0.504	0.156	0.773–0.324
MIM	0.296	0.100	0.571–0.213	0.301	0.102	0.487–0.153	0.521	0.144	0.730–0.211	0.518	0.123	0.720–0.364
RadFormation	0.346	0.161	0.784–0.222	0.357	0.066	0.543–0.257	0.577	0.149	0.715–0.236	0.559	0.145	0.804–0.334
Radiotherapy AI	0.350	0.178	0.825–0.192	**0.455**	0.094	0.642–0.320	**0.674**	0.190	0.875–0.251	**0.662**	0.179	0.840–0.361
Siemens AIRC	0.308	0.111	0.578–0.191	0.344	0.079	0.554–0.200	0.519	0.130	0.666–0.221	0.497	0.107	0.706–0.330
Therapanacea	**0.362**	0.179	0.845–0.216	0.428	0.088	0.609–0.290	0.555	0.142	0.750–0.237	0.575	0.118	0.758–0.349

Maximum difference	0.066			0.155			0.172			0.165		
	Optic chiasm (set 1 (n = 10))			Left optic nerve (set 1 (n = 10))			Right optic nerve (n = 11)			Spinalcord (set 1 (n =10))		
	Mean	STD (±)	Range	Mean	STD (±)	Range	Mean	STD (±)	Range	Mean	STD (±)	Range
Limbus AI v1.6	0.335	0.138	0.492–0.000	0.412	0.136	0.552–0.072	0.463	0.154	0.779–0.150	0.436	0.124	0.575–0.194
Limbus AI v1.5	0.333	0.080	0.445–0.162	0.412	0.136	0.551–0.072	0.463	0.152	0.768–0.150	0.411	0.133	0.578–0.170
MIM	0.303	0.076	0.474–0.219	0.266	0.088	0.389–0.073	0.323	0.116	0.623–0.169	0.467	0.114	0.587–0.223
RadFormation	0.386	0.143	0.521–0.000	0.319	0.078	0.451–0.163	0.378	0.110	0.669–0.202	0.472	0.109	0.629–0.294
Radiotherapy AI	**0.468**	0.375	0.920–0.085	**0.648**	0.205	0.914–0.378	**0.651**	0.225	0.901–0.352	**0.549**	0.070	0.641–0.465
Siemens AIRC	0.264	0.146	0.473–0.000	0.398	0.132	0.536–0.075	0.362	0.104	0.520–0.145	0.249	0.107	0.425–0.046
Therapanacea	0.346	0.064	0.453–0.258	0.398	0.118	0.532–0.103	0.451	0.143	0.746–0.164	0.509	0.082	0.632–0.366

Maximum difference	0.204			0.383			0.328			0.299		
	Left parotid gland (n = 10)			Right parotid gland (n = 9)			Left submandibular gland (n = 5)			Right submandibular gland (n =4)		
	Mean	STD (±)	Range	Mean	STD (±)	Range	Mean	STD (±)	Range	Mean	STD (±)	Range
Limbus AI v1.6	0.475	0.105	0.612–0.325	0.508	0.088	0.624–0.351	0.599	0.048	0.656–0.541	0.578	0.019	0.601–0.555
Limbus AI v1.5	0.473	0.104	0.609–0.322	0.507	0.087	0.622–0.350	0.601	0.058	0.685–0.541	0.589	0.026	0.614–0.553
MIM	0.406	0.102	0.524–0.250	0.397	0.082	0.487–0.298	0.502	0.094	0.567–0.349	0.426	0.136	0.528–0.227
RadFormation	0.409	0.091	0.533–0.273	0.406	0.120	0.556–0.241	0.603	0.050	0.672–0.543	0.611	0.035	0.655–0.570
Radiotherapy AI	**0.518**	0.123	0.666–0.324	**0.520**	0.129	0.687–0.339	**0.735**	0.170	0.861–0.503	**0.807**	0.110	0.878–0.644
Siemens AIRC	0.406	0.113	0.522–0.207	0.438	0.109	0.567–0.307	0.586	0.067	0.646–0.490	0.600	0.086	0.655–0.473
Therapanacea	0.470	0.116	0.654–0.294	0.497	0.113	0.643–0.315	0.593	0.045	0.646–0.538	0.534	0.082	0.605–0.440

Maximum difference	0.113			0.123			0.233			0.382		
	Oesophagus (set 1 (n = 6))			Oesophagus (set 3 (n = 2))			Optic chiasm (set 2 ) **			Left optic nerve (set 2) **		
	Mean	STD (±)	Range	Mean	STD (±)	Range	Mean			Mean		
Limbus AI v1.6	0.602	0.045	0.655–0.525	0.487	0.014	0.496–0.477	0.316			0.492		
Limbus AI v1.5	****	****	****	****	****	****	0.362			0.508		
MIM	***	***	***	***	***	***	0.288			0.409		
RadFormation	0.478	0.079	0.570–0.391	0.447	0.059	0.489–0.405	0.325			0.450		
Radiotherapy AI	**0.677**	0.077	0.782–0.582	**0.586**	0.048	0.620–0.553	**0.829**			***		
Siemens AIRC	***	***	***	0.463	0.061	0.507–0.420	***			0.349		
Therapanacea	0.653	0.064	0.725–0.541	0.446	0.019	0.459–0.433	0.283			**0.560**		
Maximum difference	0.199			0.140			0.546	0.211				

Bold underline values indicate the sDSC values

* No available model from AI system

** Result from only 1 tested case

*** No contours produced by AI system

**** Corresponding organ was not included to be contour by AI system in template

**Table 7:** Surface Dice Similarity Coefficient (DSC) values between manual contours and individual automated contours of OARs considered

	Left femoral head (set 1 (n =5))			Left femoral head (set 2 (n = 5))			Right femoral head (set 1 (n = 5))			Right femoral head (set 2 (n = 5))		
	Mean	STD (±)	Range	Mean	STD (±)	Range	Mean	STD (±)	Range	Mean	STD (±)	Range
Limbus AI v1.6	0.582	0.079	0.666–0.488	0.632	0.042	0.683–0.583	0.580	0.085	0.647–0.482	0.580	0.103	0.691–0.411
Limbus AI v1.5	0.582	0.079	0.666–0.489	0.633	0.042	0.683–0.586	0.581	0.085	0.648–0.484	0.582	0.103	0.691–0.412
MIM	*	*	*	0.447	0.062	0.525–0.371	*	*	*	0.394	0.173	0.544–0.112
RadFormation	0.273	0.057	0.361–0.212	0.285	0.028	0.321–0.259	0.271	0.033	0.326–0.239	0.231	0.130	0.313–0.000
Siemens AIRC	0.606	0.071	0.712–0.528	0.553	0.037	0.582–0.488	0.611	0.063	0.719–0.557	0.542	0.116	0.569–0.351
Therapanacea	**0.639**	0.086	0.741–0.543	**0.679**	0.055	0.719–0.584	**0.631**	0.076	0.706–0.535	**0.653**	0.152	0.781–0.407

Maximum difference	0.365			0.394			0.361			0.421		
	Left kidney (set 1 (n = 4))			Right kidney (set 1 (n = 4))			Heart (n = 7)			Spinalcord (set 2 (n = 9))		
	Mean	STD (±)	Range	Mean	STD (±)	Range	Mean	STD (±)	Range	Mean	STD (±)	Range
Limbus AI v1.6	0.541	0.092	0.639–0.445	0.616	0.134	0.779–0.450	0.359	0.118	0.475–0.203	0.291	0.143	0.486–0.000
Limbus AI v1.5	**0.549**	0.094	0.631–0.449	0.602	0.131	0.722–0.423	0.358	0.113	0.475–0.211	0.286	0.145	0.486–0.000
MIM	0.515	0.094	0.623–0.399	0.519	0.024	0.544–0.488	0.319	0.126	0.493–0.145	0.296	0.119	0.525–0.111
RadFormation	0.507	0.138	0.690–0.363	0.583	0.181	0.760–0.341	**0.385**	0.155	0.614–0.171	**0.354**	0.133	0.563–0.181
Siemens AIRC	0.451	0.147	0.616–0.260	0.499	0.119	0.568–0.321	0.311	0.164	0.525–0.083	0.256	0.164	0.594–0.074
Therapanacea	0.539	0.130	0.697–0.396	**0.625**	0.142	0.790–0.499	0.345	0.164	0.525–0.102	0.315	0.161	0.571–0.096

Maximum difference	0.098			0.126			0.073			0.098		
	Left lung (set 1 (n = 8))			Right lung (set 1 (n = 8))			Stomach (n = 4)			Oesophagus (set 2 (n = 4))		
	Mean	STD (±)	Range	Mean	STD (±)	Range	Mean	STD (±)	Range	Mean	STD (±)	Range
Limbus AI v1.6	0.689	0.055	0.763–0.616	0.695	0.113	0.804–0.465	**0.462**	0.080	0.581–0.417	0.472	0.052	0.534–0.421
Limbus AI v1.5	0.686	0.075	0.764–0.540	0.689	0.107	0.798–0.527	0.462	0.080	0.581–0.417	0.491	0.053	0.539–0.415
MIM	0.559	0.076	0.687–0.452	0.560	0.075	0.658–0.433	***	***	***	0.361	0.026	0.391–0.335
RadFormation	0.617	0.118	0.741–0.462	0.638	0.154	0.796–0.346	0.377	0.083	0.444–0.258	0.404	0.057	0.486–0.356
Siemens AIRC	0.628	0.076	0.699–0.506	0.636	0.082	0.709–0.497	***	***	***	0.467	0.056	0.511–0.387
Therapanacea	**0.719**	0.072	0.857–0.633	**0.709**	0.104	0.800–0.483	0.457	0.108	0.547–0.304	**0.504**	0.064	0.581–0.425

Maximum difference	0.160			0.149			0.086			0.143		
	Liver (set 1 (n = 4))			Liver (set 2 (n = 2))			Rectum (set 1 (n = 5))			Rectum (set 2 (n = 5))		
	Mean	STD (±)	Range	Mean	STD (±)	Range	Mean	STD (±)	Range	Mean	STD (±)	Range
Limbus AI v1.6	**0.582**	0.048	0.649–0.536	**0.529**	0.063	0.573–0.484	0.378	0.165	0.534–0.127	0.435	0.092	0.577–0.326
Limbus AI v1.5	0.571	0.054	0.629–0.502	****	****	****	0.376	0.165	0.532–0.125	0.436	0.092	0.578–0.328
MIM	0.420	0.053	0.489–0.371	0.439	0.074	0.492–0.387	*	*	*	0.327	0.058	0.416–0.265
RadFormation	0.420	0.079	0.496–0.311	0.484	0.067	0.531–0.437	0.356	0.160	0.495–0.085	0.405	0.027	0.438–0.370
Siemens AIRC	0.407	0.111	0.520–0.254	0.405	0.041	0.434–0.376	0.304	0.115	0.425–0.163	0.373	0.093	0.492–0.284
Therapanacea	0.465	0.100	0.581–0.337	0.513	0.079	0.569–0.457	**0.414**	0.215	0.632–0.130	**0.448**	0.050	0.501–0.389

Maximum difference	0.175			0.124			0.110			0.121		
	Bladder (set 1 (n = 5))			Bladder (set 2 (n = 5))			Left kidney (set 2) **	Right kidney (set 2) **	Left lung (set 2) **	Right lung (set 2) **		
	Mean	STD (±)	Range	Mean	STD (±)	Range	Mean	Mean	Mean	Mean		
Limbus AI v1.6	0.699	0.083	0.787–0.578	**0.602**	0.037	0.663–0.565	0.578	0.572	0.274	0.345		
Limbus AI v1.5	**0.699**	0.082	0.787–0.579	0.602	0.037	0.663–0.566	****	****	****	****		
MIM	*	*	*	0.511	0.062	0.552–0.409	*	*	***	***		
RadFormation	0.657	0.124	0.770–0.455	0.552	0.067	0.632–0.471	**0.634**	**0.607**	**0.394**	**0.475**		
Siemens AIRC	0.485	0.144	0.603–0.235	0.400	0.078	0.511–0.331	0.543	0.532	0.274	0.335		
Therapanacea	0.695	0.069	0.772–0.583	0.555	0.044	0.604–0.499	0.607	0.600	0.346	0.439		
Maximum difference	0.214			0.202			0.090	0.075	0.120	0.140		

Bold underline values indicate the sDSC values

*No available model from AI system

**Result from only 1 tested case

***No contours produced by AI system

***Corresponding organ was not included to be contour by AI system in template

## Results

The performance of each individual AI-based auto-contouring system in contouring twenty three different organs at risks considered in various clinical cases (head and neck, brain, lung, breast, pelvis, and abdomen) was quantitatively evaluated by calculating the DSC, HD and sDSC between contours of each tested organ contoured manually by expert (Manual) and automatically by each software, Radiotherapy AI (RTAI), Limbus AI version 1.5 (Lim1.5) and version 1.6 (Lim1.6), Therapanacea (TH), MIM (MIM), Siemens AIRC (SAIRC) and RadFormation (RF). The higher DSC, sDSC and lower HD value illustrate better agreement with the Manual. The mean, standard deviation, range, and maximum absolute difference of the DSC for each considered OAR case in head and neck and brain cases are illustrated in Table 2. Similarly, the values for lung, breast, pelvis, and abdomen cases are presented in Table 3. The mean, standard deviation, range, and maximum absolute difference of the maximum HD for each considered OAR case in head and neck and brain cases are illustrated in Table 4. Similarly, the values for lung, breast, pelvis, and abdomen cases are presented in Table 5. The mean, standard deviation, range, and maximum absolute difference of the surface DSC for each considered OAR case in head and neck and brain cases are illustrated in Table 6. Similarly, the values for lung, breast, pelvis, and abdomen cases are presented in Table 7. Both highest mean DSC and sDSC values and lowest HD value for each case are presented in bold and highlighted. The distribution of individual data for each OAR were tabulated and illustrated in both scatter and box plot, corresponding statistical results are illustrated in Supplementary data A for DSC, B for HD, C for sDSC. The box plot of data for individual AI systems for all considered OARs are shown in Supplementary data 1 (DSC), 2 (HD), 3 to 6 (sDSC with different tau value).

## Discussion

In this study, seven different AI-based auto-contouring systems were tested to study each system’s performance in contouring organs at risk considered in different clinical cases. In general, the study showed sDSC values were considerably smaller than volumetric DSC values, especially for OARs with large volumes as reported from previous studies [10, 21, 27].

In head and neck and brain cases, the contours delineated by each AI system showed good agreement with reference contours for most of OARs considered. The DSC for brain, brainstem, left eye, right eye, left parotid gland, right parotid gland, left submandibular gland and right submandibular gland from tested AI systems were comparable to the previous study by Doolan *et. al* [10] and by Liu *et. al* [19]. This study reported slightly lower sDSC for brain, brainstem, left eye, right eye, left parotid gland, right parotid gland, left submandibular gland and right submandibular gland from tested AI systems [10]. The HD for the same set of OARs from tested AI systems were slightly higher compared to previously reported HD [10].

The study found that the AI systems had shown reduced and inconsistent performance in contouring small and complex structures such as optic structures and oesophagus which is difficult to visualise in CT images rather than MR. The reduced and inconsistent performance of auto contouring systems in contouring small and complex structures had been previously reported in other studies. The previous study by Liu *et. al* [19] reported low DSC value for optic chiasm and wide variation in DSC value for the left and right optic nerve across multiple previous studies. Similarly, the reduced and inconsistent performance was found in this study for oesophagus cases which correlates with previously reported DSC, sDSC and HD values for oesophagus case [10].

The Radiotherapy AI software showed the best performance across all tested systems. The better agreement between the Radiotherapy AI contours and manual contours in this study may be due to the fact that the Radiotherapy AI model was trained on our clinic’s contours and therefore produced contours similar to those used in our clinic. This result demonstrates the advantages of an in-house built AI system or AI systems which were trained based on clinic-specific data. This would provide contours more similar to those currently used in that clinic. On the other hand, this could perpetuate incorrect contouring and does not provide review of current contouring practice. Nor would it lead to standardisation of contours across radiation therapy centres. However, the study found very small maximum differences in both DSC and HD values across all tested systems. So, in most test cases, the shape of contours delineated by AI systems were comparable to each other.

Low DSC of spinal cord was found across all tested AI systems during this study where previously reported DSC of spinal cord was considerably higher [10, 19]. This large disagreement occurs because the manual contours only cover the part of spinal cord which lies in the treatment field, while AI systems contour all area of spinal cord in the image as shown in Fig. 1.

There was no specific AI based software showing overall superior performance compared to others in lung, breast, pelvis and abdomen cases. Again, the very small maximum differences in both DSC and HD values across all tested systems supports that the shapes of contours delineated by each AI system are comparable to each other.

The DSC for bladder, left and right lungs, heart, left and right kidneys, liver, rectum and stomach from tested AI systems were comparable to the previous study [1, 10]. This study reported slightly lower sDSC for bladder, heart, left and right lung, liver from tested AI systems compared to previously reported sDSC [10]. The HD for same set of OARs from tested AI systems were slightly higher compared to previously reported HD [10]. This study reported slightly lower performance in rectum case compared to previously reported DSC, sDSC and HD [10].

Both left and right femoral head DSC and sDSC were comparable and HD was slightly higher compared to previously reported DSC, sDSC and HD [10]. The study found that DSC values of RadFormation were lower and HD values were higher compared to other tested AI for both left and right femoral head cases. The low DSC values, high HD values and large variation in the average DSC value when compared with other AI software were due to the difference in contouring method of RadFormation, which delineated the femoral head only while other systems and the manual reference contours included a small portion of the femoral neck as shown in Fig. 2.

There were several limitations in this study. Firstly, there were limitations in a few tested AI systems’ models. The Radiotherapy AI model was only available for head and neck, and brain regions, while the MIM model only contoured structures in male pelvis cases at the time of study. Not long after the analysis of the study was performed, most AI systems updated their models to improve their contouring quality and also offered additional structures to be contoured. Due to the rapid development of the field, it was not feasible to reflect the performance of all tested AI systems up to date. So it must be noted to the reader that this study only reflects the specific version of each tested system which was stated previously in the method section. This implies that clinics, whether in the planning stages of implementing or already having integrated an AI system, require a set of workflows or a tool to assess the AI system’s performance. This will be crucial for keeping pace with the rapid advancements in this field. Secondly, the sample size used may have been insufficient to provide adequate power for the statistical tests [30]. The sample size for some OARs was very small, with only four or five reference contours for the right submandibular gland and the stomach. So the statistical test performed for data sets with less than five samples were ignored and denoted as ***** in supplementary data A, B and C. Thirdly, in a few cases, some software systems were not able to produce particular contours for every patient. For instance, the Radiotherapy AI produced an incompleted contour of the left optic nerve by contouring on only a single CT image slice in case HN10. Fourthly, the manual contours considered as the reference during this study were contoured by only a single expert. Using cross-validated contours would have ensured the accuracy of the reference data. Lastly, Baroudi *et. Al* [3] discussed that to clinically accept the automated contours, the AI systems need to be evaluated in multiple domains such as quantitative evaluation of automated contours using geometric metrics, qualitative evaluation of automated contours by the end users using Likert scales and Turing tests, the dosimetric evaluation of automated contours by assessing the impact on the dose for OARs and targets when automated contours were used in planning, and lastly assessing the improvement of efficiency of clinical workflow when the AI system was used. This study exclusively conducted a quantitative evaluation of automated contours and as one of the main intentions of this study was to provide a starting point or guidance to other clinics that are considering implementing the AI system into their clinical workflow, additional forms of evaluations are planned as future work.

## Conclusion

The study successfully investigated the performance of multiple AI-based auto-contouring systems by performing quantitative comparisons. Each tested AI system was able to produce comparable contours to the expert’s contours of organs at risk which implies that these contours can potentially used for clinical use after experts’ assessment and QA on the system. This study has demonstrated a method of comparing contouring software options which could be replicated in clinics or used for ongoing quality assurance of purchased systems. A statically significant difference between AI systems’ performance in various cases was found, but the absolute difference between values was not large which illustrate that all tested AI systems’ performance were comparable to each other. A reduced performance of AI systems in the case of small and complex anatomical structures was found and reported, showing that it is still essential to review each contour produced by AI systems for clinical uses.

**Supplementary information** There are nine supplementary files that contain all results sets collected during the study.

Supplementary file 1 and 2 contains box plot of all DSC (Supplementary data 1_DSC Box plot) and HD (Supplementary data 2_HD Box plot) data for each tested AI based contouring system. and Supplementart file 3 to 6 contains box plot of all sDSC with different $\tau$ value applied (0 to 3 mm) data for each tested AI based contouring system.

Supplementary file 7 (Supplementary data A_DSC) contains all results data for each tested organ at risk obtained from the method conducted in this study. Each tab with the name of the organ at risk tested has:

- The table of calculated dice similarity coefficient
- The scatter plot and box plot of data
- Histogram, Q-Q plot and table of Shapiro-Wilk Test results
- The table of statistical test results

Supplementary file 8 (Supplementary data B_HD) contains all results data for each tested organ at risk obtained from the method conducted in this study. Each tab with the name of the organ at risk tested has:

- The table of calculated maximum Hausdorff distance
- The scatter plot and box plot of data
- Histogram, Q-Q plot and table of Shapiro-Wilk Test results
- The table of statistical test results

Supplementary file 9 (Supplementary data C_sDSC) contains all results data for each tested organ at risk obtained from the method conducted in this study. Each tab with the name of the organ at risk tested has:

- The table of calculated surface dice similarity coefficient with different $\tau$ value applied (0 to 3 mm)
- The scatter plot and box plot of data
- Histogram, Q-Q plot and table of Shapiro-Wilk Test results
- The table of statistical test results

## Supplementary Information

Below is the link to the electronic supplementary material.

Supplementary file 1 (xlsx 4242 KB)

Supplementary file 2 (xlsx 4166 KB)

Supplementary file 3 (xlsx 15632 KB)

Supplementary file 4 (xlsx 861 KB)

Supplementary file 5 (xlsx 838 KB)

Supplementary file 6 (xlsx 960 KB)

Supplementary file 7 (xlsx 996 KB)

Supplementary file 8 (xlsx 922 KB)

Supplementary file 9 (xlsx 876 KB)

## Data Availability

The patient data that support the findings of this study are available on request from the corresponding author but will be subject to ethics and hospital approval. The data are not publicly available due to privacy restrictions. All calculated values are available on request.
